# Supplementary material for: PAH-pollution effects on sensitive and resistant embryos: Integrating structure and function with gene expression
Source: PLoS One. 2021 Apr 6;16(4):e0249432. doi: 10.1371/journal.pone.0249432 (PMC8023486; doi:10.1371/journal.pone.0249432)
Supplement: Attachment — Submitted filename: Rebuttal_Letter_Response_to_Reviewers_Comments.docx [file pone.0249432.s002.docx]

Dear Dr. Cao and the reviewers,

Thank you for your valuable comments and suggestions which strengthen the content and relevance of our manuscript. Please consider our responses below in red.

*1. Please ensure that your manuscript meets PLOS ONE's style requirements, including those for file naming. The PLOS ONE style templates can be found at*

[*https://journals.plos.org/plosone/s/file?id=wjVg/PLOSOne_formatting_sample_main_body.pdf*](https://urldefense.com/v3/__https:/journals.plos.org/plosone/s/file?id=wjVg*PLOSOne_formatting_sample_main_body.pdf__;Lw!!Mih3wA!XzDf3Ean7QFoQ6N3YORaQb3npbWZrLJtAJq-2v5Pbnj_bPtgItFALjN7bCKLw9B_RyM$) *and*

[*https://journals.plos.org/plosone/s/file?id=ba62/PLOSOne_formatting_sample_title_authors_affiliations.pdf*](about:blank)

We have modified the manuscript to meet PLOS ONE’s style requirements.

*2. In your Methods section, please provide additional information regarding the permits you obtained for the work. Please ensure you have included the full name of the authority that approved the field site access and, if no permits were required, a brief statement explaining why.*

We now state in the manuscript that no permits were required for site access or sampling (line 105-106 and 144-147): “No permission was required for sampling sites access and sample collection.” “Fish collection was completed within publicly available lands. No permission was required for sampling sites access or to collect fish for non-commercial purposes, as *F. heteroclitus* does not have endangered or protected status and does not require collecting permits.”

*3. In your Methods section, please include a comment about the state of the animals following this research. Were they released, euthanized or housed for use in further research? If any animals were sacrificed by the authors, please include the method of euthanasia and describe any efforts that were undertaken to reduce animal suffering.*

We now clearly state that “Adult fish were housed in the lab for use in further research.” (line 154).

*4. We note that you are reporting an analysis of a microarray, next-generation sequencing, or deep sequencing data set. PLOS requires that authors comply with field-specific standards for preparation, recording, and deposition of data in repositories appropriate to their field. Please upload these data to a stable, public repository (such as ArrayExpress, Gene Expression Omnibus (GEO), DNA Data Bank of Japan (DDBJ), NCBI GenBank, NCBI Sequence Read Archive, or EMBL Nucleotide Sequence Database (ENA)). In your revised cover letter, please provide the relevant accession numbers that may be used to access these data. For a full list of recommended repositories, see* [*http://journals.plos.org/plosone/s/data-availability#loc-omics*](about:blank#loc-omics) *or* [*http://journals.plos.org/plosone/s/data-availability#loc-sequencing*](about:blank#loc-sequencing)*.*

As provided in the revised cover letter, our data were deposited in NCBI’s Gene Expression Omnibus and are accessible through GEO Series accession number GSE130754.

*5. Thank you for including your ethics statement:  "All experiments were performed according to approved protocols per Institutional Animal Care and Use Committees, North Carolina State University and Duke University.".*

*Please amend your current ethics statement to confirm that your named ethics committee specifically approved this study.*

*For additional information about PLOS ONE submissions requirements for ethics oversight of animal work, please refer to* [*http://journals.plos.org/plosone/s/submission-guidelines#loc-animal-research*](https://urldefense.com/v3/__http:/journals.plos.org/plosone/s/submission-guidelines*loc-animal-research__;Iw!!Mih3wA!XzDf3Ean7QFoQ6N3YORaQb3npbWZrLJtAJq-2v5Pbnj_bPtgItFALjN7bCKLLXxvrLw$)

*Once you have amended this/these statement(s) in the Methods section of the manuscript, please add the same text to the “Ethics Statement” field of the submission form (via “Edit Submission”).*

Our ethics statement has been amended to “Experimental procedures for this study were approved by Institutional Animal Care and Use Committee (IACUC) at North Carolina State University (IACUC assurance A3331-01), and non-surgical tissue sampling and fish embryo culturing and maintenance was approved by the IACUC at North Carolina State University (IACUC assurance A3331-01) and the Duke University (IACUC assurance A3195-01).” (line 155-159) and added to the “Ethics Statement” field of the submission form.

***Comments to the Author*** *1. Is the manuscript technically sound, and do the data support the conclusions?

The manuscript must describe a technically sound piece of scientific research with data that supports the conclusions. Experiments must have been conducted rigorously, with appropriate controls, replication, and sample sizes. The conclusions must be drawn appropriately based on the data presented.*

*Reviewer #1: Yes*

*Reviewer #2: Yes*

*2. Has the statistical analysis been performed appropriately and rigorously?*

*Reviewer #1: Yes*

*Reviewer #2: No*

*3. Have the authors made all data underlying the findings in their manuscript fully available?

The* [*PLOS Data policy*](https://urldefense.com/v3/__http:/www.plosone.org/static/policies.action*sharing__;Iw!!Mih3wA!XzDf3Ean7QFoQ6N3YORaQb3npbWZrLJtAJq-2v5Pbnj_bPtgItFALjN7bCKLzVZx7zw$) *requires authors to make all data underlying the findings described in their manuscript fully available without restriction, with rare exception (please refer to the Data Availability Statement in the manuscript PDF file). The data should be provided as part of the manuscript or its supporting information, or deposited to a public repository. For example, in addition to summary statistics, the data points behind means, medians and variance measures should be available. If there are restrictions on publicly sharing data—e.g. participant privacy or use of data from a third party—those must be specified.*

*Reviewer #1: Yes*

*Reviewer #2: Yes*

*4. Is the manuscript presented in an intelligible fashion and written in standard English?

PLOS ONE does not copyedit accepted manuscripts, so the language in submitted articles must be clear, correct, and unambiguous. Any typographical or grammatical errors should be corrected at revision, so please note any specific errors here.*

*Reviewer #1: Yes*

*Reviewer #2: Yes*

*5. Review Comments to the Author

Please use the space provided to explain your answers to the questions above. You may also include additional comments for the author, including concerns about dual publication, research ethics, or publication ethics. (Please upload your review as an attachment if it exceeds 20,000 characters)*

*Reviewer #1: In this manuscript, the author chose the F. heteroclitus embryos from the Elizabeth River Superfund population and the relatively unpolluted population nearby to simulate the sensitive and resistant of the natural population, and exposed them to contaminated and clean sediment extracts to explore the mechanism of POP population differences through the detection of morphology, physiology, chemical absorption and gene expression changes of embryos at different key developmental stages. Starting from developmental toxicity, this article combined with actual environmental exposure, which is an interesting and meaningful study.
The article is well written. However, the explanation of the exposure dose of the extract in this study is not very clear. Is the composition of the extract at different times or seasons consistent? Explaining these clearly helps prove the significance of the research.*

Since all sediment samples were collected in April 2007 during a single sampling event, the variation in dosing is not addressed. We now clearly state the single-season sampling event (line 97-100); relevant sediment chemical composition is presented in Figure 1A-B, and we added a supplementary table (S1 Table) that lists all PAHs tested and corresponding concentrations; body burdens of exposed embryos are shown in Figure 1C.

*Regarding the follow-up of microarray, the author is hoped to add the results verification of the differentially expressed genes, which would appear more solid and credible.*

We appreciate the comment about verifying the microarray data with qRT-PCR assay. There is, however, an experimental justification in certain instances that indicate such validation is not necessary. Since biological replication and reproducibility of results is the aim of the microarray experimental design, a well-balanced design with sufficient biological replication for array data analyses yields a statistical power for differential gene expression detection. Such microarray platforms with rigorous statistical analysis are sufficient to not warrant technical replicates. Moreover, if the same samples are analyzed twice but only with a different technology (microarray vs. qRT-PCR), the results would serve as a technical validation, possibly revealing a "technology bias". Lastly, microarrays vs. qRT-PCR may be relevant when the “true” fold-changes are a desired outcome, and the arrays were used as a screening tool. Analyzing different samples with the same technique will provide an estimate of the biological and technical variance. Analyzing different samples with a different technique will reveal a combination of "technology bias" and the "between-assay variance". Our study focuses on identifying differential expression, while accurate measurement of fold-changes, which is required for setting up quantitative dynamic models of gene-regulation or other molecular-physiology processes, is not the goal of our study. We respectfully include three references addressing the questions of necessity of microarray data validation relative to sufficient biological replication and balanced experimental design.

Chuaqui, R. F., Bonner, R. F., Best, C. J., Gillespie, J. W., Flaig, M. J., Hewitt, S. M., ... & Emmert-Buck, M. R. (2002). Post-analysis follow-up and validation of microarray experiments. *Nature genetics*, *32*(4), 509-514.

Morey, J. S., Ryan, J. C., & Van Dolah, F. M. (2006). Microarray validation: factors influencing correlation between oligonucleotide microarrays and real-time PCR. *Biological procedures online*, *8*(1), 175-193.

Wise, R. P., Moscou, M. J., Bogdanove, A. J., & Whitham, S. A. (2007). Transcript profiling in host–pathogen interactions. *Annu. Rev. Phytopathol.*, *45*, 329-369.

*Reviewer #2: The present study chemically characterized clean and heavily polluted sites and exposed fish embryos to PAH polluted sediment extracts during four critical developmental stages, and used gene expression analysis for different developmental to identify differentially expressed genes which helps to explain mechanisms of sensitivity and resistance to polluted environments during vertebrate animal development. The manuscript is generally well prepared and the results are informative, and I have some comments to improve the ms based on its present form.*

*Title: the primary pollutants investigated in the study are polycyclic aromatic hydrocarbons, which should be reflected in the title. Currently the title does not identify what pollutants are the pollutants of interest.*

We have added “PAH” to the title to reflect the focus of the study. The title now reads “PAH-pollution effects on sensitive and resistant embryos: Integrating structure and function with gene expression” (line 3).

*The results of gene expression should consider the issue of multiple testing adjustment. It is possible that many of the identified genes are just false positives simply because of the high number of genes (n=6769) being tested. May consider False Discovery Rate, Bonferroni correction or other similar methods.*

We agree with the comment and updated Table 2 to show the number of significant genes after False Discovery Rate filtration (pFDR < 0.05) is applied. We modified the manuscript accordingly to address the false positive issue in line 602-605 and 615-616. We also reinforced the original statement in the Methods “Using this p-value reveals more genes that may be differentially expressed but risks identifying genes that may be false positives.” (line 351-352) with “False discovery rate (pFDR < 0.05) was also used for filtering residuals.” (line 352-353)

*It is unclear how many kinds of PAHs were tested in the study, and would better provide a list of analyzed individual PAHs in the methods section (along with their molecular weights and low/high molecular weight designation), either in text or a supplemental table to facilitate the understanding.*

We appreciate the comment and now list all PAHs tested in the study, including their molecular weights, in S1 Table. We also clarified the designation of low (fewer than 4 aromatic rings) and high (4 or more aromatic rings) molecular weight PAHs (line 359-361).

*Fig 1. The figure legend explained that “Low concentrations of only 10 of the 42 PAHs tested, mostly low molecular weight naphthalenes, are detected at the reference site”, however, the B panel showed no blue columns for the reference site. Please check.*

For Fig 1B, the concentrations of PAHs detected at the reference site are significantly lower (non-zero concentrations are mostly below 10 ng/L) than those for Elizabeth River and are thus not visible on the figure. We have adjusted the axis so the PAH with the highest concentration (C1 - Naphthalenes, 11.86 ng/L) is more visible. This issue is also addressed in line 370-373 and 386-387 and further detailed in S1 table.

*Fig 2. Please indicate what is the meaning of the asterisk as shown in the figure. There is no explanation for the asterisk in the legend. Also be consistent to use ‘one-way ANOVA’ and ‘1-way ANOVA’ and related p values for cutoff (<0.05 vs. <0.01).*

We much appreciate this comment and have modified the figure so that the statistical significance is more clearly illustrated. We now explain the meaning of the asterisk in line 438-439 (“*, p < 0.05; **, p < 0.01; ***, p < 0.001; ****, p < 0.0001.”). We also changed “1-way ANOVA” to “one-way ANOVA” (line 438).

*Fig 3. The issue of asterisk also exists here. Please explain the meaning of R2 in the C panel and how to calculate it, also explain the meaning of the green curves in the same panel.*

We have modified the figure so that the statistical significance is more clearly illustrated and now explain the meaning of the asterisk in line 496 (“****, p < 0.0001.”). The R2 and regression line are now explained in line 495-497.
